# Pre- and post-chemotherapy spermatogenesis in male patients with malignant bone and soft tissue tumors

**DOI:** 10.3389/fphar.2023.1324339

**Published:** 2023-12-06

**Authors:** Teppei Takeshima, Noboru Mimura, Shun Aoki, Tomoki Saito, Jurii Karibe, Kimitsugu Usui, Shinnosuke Kuroda, Mitsuru Komeya, Yasushi Yumura

**Affiliations:** ^1^ Department of Urology, Reproduction Center, Yokohama City University Medical Center, Yokohama, Kanagawa, Japan; ^2^ Department of Urology, Saiseikai Yokohama City Nanbu Hospital, Yokohama, Kanagawa, Japan; ^3^ Department of Urology, Sagami Rinkan Hospital, Sagamihara, Kanagawa, Japan; ^4^ Department of Urology, Kanagawa Cancer Center, Yokohama, Kanagawa, Japan; ^5^ Glickman Kidney and Urological Institute, Cleveland Clinic Foundation, Cleveland, OH, United States; ^6^ Department of Urology, Yokohama City University Hospital, Yokohama, Kanagawa, Japan

**Keywords:** sperm, sperm cryopreservation, malignant bone tumor, malignant soft tissue tumor, sarcoma, fertility preservation, ifosfamide

## Abstract

**Introduction:** Malignant bone and soft tissue tumors, commonly called sarcomas, predominantly originate in bone and soft tissues and typically affect individuals at a younger age. Following the resection of the primary tumor, treatment often necessitates radiation therapy and gonadotoxic chemotherapy, the specifics of which depend on the disease’s stage Conversely, there is a notable concern regarding the potential loss of fertility due to these treatments. Consequently, it is recommended that men consider sperm cryopreservation before initiating treatment. This study aims to assess spermatogenesis in male patients diagnosed with malignant bone and soft tissue tumors before and after chemotherapy.

**Methods:** This study involved 34 male patients diagnosed with malignant bone and soft tissue tumors and subsequently underwent sperm cryopreservation before initiating treatment. Medical records included details about the primary disease, age, marital status at presentation, semen analysis results, treatment regimen and number of courses, post-treatment semen analysis, renewal status and outcomes.

**Results:** The mean age at the time of sperm cryopreservation was 22.8 years. The median semen volume was 2.5 mL, sperm concentration was 32.6 million/ml, and sperm motility was 38.5%. Following chemotherapy, semen analysis was conducted on 12 patients, with ifosfamide being the predominant drug used in all cases. Among these 12 patients, eight retained viable spermatozoa, and two successfully achieved spontaneous pregnancies resulting in live births. In one of the remaining four cases where no sperm were detected in ejaculate, a live birth was achieved through intracytoplasmic sperm injection using cryopreserved sperm.

**Discussion:** While ifosfamide, the primary chemotherapy drug for patients with malignant bone and soft tissue tumors, was associated with severe impairments in spermatogenesis, recovery of spermatogenesis was observed in many cases. However, there were instances of prolonged azoospermia. Even in such cases, assisted reproduction using cryopreserved sperm remained viable for achieving parenthood. In light of these findings, offering patients the opportunity for fertility preservation is advisable.

## 1 Introduction

Malignant bone and soft tissue tumors (BSTT), known as sarcomas, represent rare non-epithelial malignancies originating in bone and various bodily tissues, including muscles, fat, blood vessels, nerves, and connective tissues. These malignancies encompass several subtypes, including osteosarcoma, chondrosarcoma, Ewing’s sarcoma, leiomyosarcoma, liposarcoma, rhabdomyosarcoma, and synovial sarcoma. These malignancies are uncommon, comprising only 1% of all malignant tumors (Siegel et al., 2022; Siegel et al., 2023) Notably, most cases of malignant BSTT manifest in younger age groups. For instance, osteosarcoma exhibits a bimodal peak of incidence in individuals in their 20s and after age 65. Osteosarcoma is the third most prevalent cancer type among children and adolescents aged 12–18, following leukemia and lymphoma.

The prognosis of these diseases has seen significant Improvement with the advent of multidisciplinary treatment approaches. The primary treatment approach is surgery; however, radiation therapy or chemotherapy may be indicated in those with more-severe malignancy grades. Notably, chemotherapy often involves alkylating agents with high testicular toxicity (Hiraga and Ozaki, 2021; Tanaka and Ozaki, 2021). Alkylating agents tend to cross-link sperm DNA double strands, impeding DNA synthesis and RNA transcription and eventually resulting in DNA breakage (Qiu et al., 1995; Ledingham et al., 2020). Damage to spermatogonia DNA within the seminiferous tubules can temporarily halt spermatogenesis, leading to azoospermia. The likelihood of permanent azoospermia or spermatogenesis restoration depends on the drug dose. For instance, doses exceeding 7.5 g/m^2^ for cyclophosphamide and 60 g/m^2^ for ifosfamide (Meistrich et al., 1992; Williams et al., 2008) increase the risk of permanent azoospermia. Use of doxorubicin-based chemotherapy regimens as alternatives to ifosfamide for malignant soft tissue tumors may induce testicular histopathological deformities, oligozoospermia, and abnormal sperm morphology. These drugs can affect the physiological role of Leydig and Sertoli cells, potentially causing chromosome abnormalities and DNA synthesis disruptions (Mohan et al., 2021a). Cisplatin (CDDP), used in osteosarcoma treatment, is associated with an increased risk of permanent azoospermia when cumulative doses exceed 400 mg/m^2^ (DeSantis et al., 1999). Given that many individuals diagnosed with malignant BSTT are young, these adverse effects can lead to the loss of opportunities for significant life events like marriage, pregnancy, and childbirth. Consequently, sperm cryopreservation emerges as a crucial facet of fertility preservation before initiating treatment, particularly for male patients, due to the elevated risk of azoospermia and severe spermatogenesis dysfunction (Oktay et al., 2018).

The American Society for Clinical Oncology (ASCO) practice guideline identifies alkylating agents as high-risk drugs for causing permanent azoospermia and advocates for sperm cryopreservation as a means of fertility preservation. Regrettably, the urgency of treatment and limited awareness of fertility preservation among orthopedic surgeons often result in missed opportunities for pretreatment fertility preservation. In such cases, the timeline for recovering spermatogenesis also becomes a critical concern. Despite these risks, no prior studies have investigated spermatogenesis following chemotherapy for malignant BSTT. Thus, the primary objective of this study is to investigate spermatogenesis function during pretreatment sperm cryopreservation and assess the recovery of spermatogenesis following gonadotoxic treatments in patients afflicted with malignant BSTT.

## 2 Materials and methods

### 2.1 Study design

This retrospective study evaluated patients with malignant BSTT who underwent sperm cryopreservation for fertility preservation at the Reproduction Center of Yokohama City University Medical Center between September 2012 and August 2023. Data extracted from medical records encompassed details such as age, marital status, disease type, planned treatment, pretreatment and post-treatment semen parameters, and the status of cryopreservation maintenance.

Furthermore, for patients who could follow-up on semen analysis during annual cryopreservation follow-ups at the outpatient clinic, we analyzed spermatogenesis recovery in ejaculate and the time required for this recovery. The appearance of sperm in the ejaculate, even if scarce, was considered a marker of successful recovery. For this retrospective observational study, patient consent was assumed, utilizing an opt-out approach. The study’s design received approval from the institutional review board of Yokohama City University Medical Center (IRB No: F211100008).

### 2.2 Semen analysis and sperm cryopreservation

Semen samples were collected through masturbation at our hospital after varying intervals of sexual abstinence. Following a 30-min liquefaction period at room temperature, semen analyses were conducted as per the World Health Organization (WHO) 2021 recommendations (World Health Organization, 2021), using the Sperm Motility Analyzing System (SMAS™: DITECT Corp., Tokyo, Japan).The freezing procedure involved diluting the semen sample with an equal volume of freezing medium (Sperm Freeze™, Kitazato, Co. Ltd., Tokyo, Japan) and transferring it into a straw tube. Both ends of the straw tube were then sealed using a sealer. These sealed straw tubes were arranged in a column suspended in vapor-phase nitrogen for 5 min before being placed in liquid nitrogen for long-term storage.

### 2.3 Maintenance and usage for cryopreserved sperm

Patients undergo an annual reservation for follow-up in the outpatient clinic and are inquired about their preference regarding the retention or disposal of cryopreserved sperm. Semen analysis is conducted upon the patient’s request to verify the presence of spermatozoa, thereby confirming spermatogenesis function.

In cases where a patient desires to discard the cryopreserved sperm or in the unfortunate event of their passing, the cryopreserved sperm is appropriately discarded. Alternatively, when the cryopreserved sperm is utilized to conceive a child, the frozen-thawed sperm is typically employed alongside intracytoplasmic sperm injection (ICSI) within our facility.

### 2.4 Data analysis

Summary statistics were computed, and the data are presented as mean values with standard deviation or median values with interquartile range (IQR) for continuous variables. The proportion of individual elements related to the total is expressed as a percentage (%).

## 3 Results

In this study, 34 patients were included, with a mean age of 22.8 years. The distribution of diseases among these patients was as follows, listed in descending order of frequency: osteosarcoma (11/34; 32.4%), synovial sarcoma (6/34; 17.6%), Ewing’s sarcoma (5/34; 14.7%), rhabdomyosarcoma (3/34; 8.8%), epithelioid sarcoma (3/34; 8.8%), leiomyosarcoma (2/34; 5.9%), and liposarcoma (2/34; 5.9%).

All patients underwent tumor resection, and the majority (32 patients) received anticancer drugs, except for one case under surveillance and one undergoing radiotherapy. Of those who received chemotherapy, 29 patients were treated with ifosfamide-based regimens, except three patients with osteosarcoma who received cisplatin-based chemotherapy. Notably, all 34 patients had successfully ejaculated sperm and were deemed eligible for sperm cryopreservation.

At the time of cryopreservation, the median semen volume was 2.45 mL, with a sperm concentration of 32.5 × 10^6^/mL and a motility rate of 38.5% (see Table 1).

**TABLE 1:** Patient characteristics.

Mean age (SD), years		22.8 (7.3)
Maritai status (%), n	married	3 (8.8)
	unmarried	31 (91.2)
Diseases (%), n	osteosarcoma	11 (32.4)
	synovial sarcoma	6 (17.6)
	Ewing’s sarcoma	5 (14.7)
	rhabdomyosarcoma	3 (8.8)
	epithlioid sarcoma	3 (8.8)
	leiomyosarcoma	2 (5.9)
	liposarcoma	2 (5.9)
	other	2 (5.9)
Median semen analysis (IQR)	semen volume, ml	2.5 (1.5, 3.6)
	sperm concentration, million/ml	32.5 (15.7, 54.5)
	sperm motility, %	38.5 (19.2, 52.7)
Regimen (%), n	ifosfamide-based	29 (85.3)
	cisplatin-based	3 (8.8)
	no chemotherapy	2 (5.9)
Cycles (%), n	1–5	21 (65.6)
	6–10	9 (28.1)
	11-	1 (3.1)
	unknown	1 (3.1)

Excluding the 7 patients who were followed for less than 1 year, the outcomes of 27 patients were as follows: death in 8, another 8 requested their frozen sperm be discarded, 7 kept their sperm under cryopreservation, and 4 were lost to follow-up. The median post-cryopreservation sperm survival for those who died was 2 years (IQR: 1–3 years), indicating that the prognosis for patients with malignant BSTT remains poor.

Among the 13 cases with available semen analysis follow-up data, 12 were examined, all of which involved ifosfamide-based chemotherapy, except for one case where chemotherapy was not administered. Spermatogenesis successfully recovered in eight cases (66.7%), with an average recovery time of 3 years (see Table 2). At the time of sperm cryopreservation, these eight cases demonstrated a median sperm concentration of 20.3 × 10^6^/mL (IQR: 17.4–27.5) and median motility of 38.5% (IQR: 14.1–45.7). For the best findings after anticancer drug administration, the median sperm concentration was 32.1 × 10^6^/mL (IQR: 11.8–44.9) and the median motility was 38.5% (IQR: 28.1–59.7) (see Table 3). Notably, six cases exhibited improved semen parameters compared to those during cryopreservation.

**TABLE 2:** Outcome of patients whose spermatogenesis could be tracked.

Case	Age, years	Key drug*	Number of cycle	Time to evaluate, years	Spermatogenesis recovery	Delivery	Follow-up duration, years	Outome
1	16	IFM	2	1	-	-	1	died
2	18	IFM	unknown	7	-	-	7	ongoing
3	21	IFM	10	3	-	+**	7	ongoing
4	27	IFM	7	1	-	-	1	lost to follow
5	16	IFM	3	7	+	-	8	discarded
6	20	IFM	8	3	+	-	7	discarded
7	20	IFM	5	2	+	-	3	discarded
8	21	IFM	12	3	+	+	7	discarded
9	22	IFM	4	3	+	-	3	discarded
10	24	IFM	7	2	+	+	6	discarded
11	26	IFM	4	2	+	-	4	discarded
12	34	IFM	5	2	+	-	5	died

* IFM, ifosfamide.

** delivery with assisted reproduction.

**TABLE 3:** Pre- and post-chmotherapy semen analysis of patients with malignant BSTT.

	Sperm recovery	Prolonged azoospermia
Patients, n	8	4
Mean age, years	22.9	20.5
Median number of cycles (range), n	5 (4–12)	7 (2–10)*
Median semen analysis		
Pre-chemotherapy		
Concentration, 10^6^/mL	20.3	28.8
Motility, %	38.5	49.5
Post-chemotherapy		
Concentration, 106/mL	32.1	0.0
Motility, %	38.5	0.0

* excluding one case with unkown number of cycles.

Additionally, among these cases, seven patients (one of whom had sadly passed due to disease progression) opted to discard their cryopreserved sperm. Remarkably, in two of these instances, the partners of the patients achieved spontaneous pregnancies and gave birth to their children (see Table 2). One of the patients had synovial sarcoma, and the other patient had Ewing’s sarcoma; their sperm concentrations were 14.5 × 10^6^/mL and 21.2 × 10^6^/mL and motilities were 40.2% and 41.9% at the time of sperm cryopreservation, respectively. After 7 and 12 courses of ifosfamide and a doxorubicin-centered regimen, spermatogenesis was recovered in 2 and 3 years. The maximum sperm concentrations of these patients were 1.8 × 10^6^/mL and 42.9 × 10^6^/mL, and the maximum motilities were 29.0% and 45.0%, respectively.

## 4 Discussion

This is the first retrospective observational study to assess pre- and post-chemotherapy spermatogenesis in patients with malignant BSTT. This issue is rarely studied as malignant BSTT is a rare condition, with a poor prognosis. Follow-ups for spermatogenesis are difficult to conduct as young patients often resist these evaluations.

Notably, the patients in this study were relatively young at the time of cryopreservation compared to individuals with other malignant diseases like testicular tumors and leukemia. Unfortunately, the prognosis remains unfavorable, with 8 out of 27 patients who could be followed for more than a year succumbing during the follow-up period.

The urgency of diagnosis and treatment often compels clinicians to initiate treatment without sufficient explanations about fertility preservation, potentially resulting in fertility loss. Some orthopedic oncologists have recommended pretreatment sperm cryopreservation for malignant BSTT patients under 45, with significant interest shown by unmarried and childless patients (Hoshi et al., 2014). Nevertheless, there is a paucity of reports summarizing spermatogenesis outcomes before and after chemotherapy for this condition.

In a previous study conducted in rabbits, a decrease in testicular weight and sperm count was observed with an increase in the dose of ifosfamide. Although prolonged spermatogenesis dysfunction was noted in response to dose, recovery was achieved by 8 weeks among rabbits who received 240 mg/kg of ifosfamide. (Ypsilantis et al., 2003). In humans, 1.5–3 g/m^2^, or 30–60 mg/kg is usually administered over 3–5 days for malignant BSTT. However, the abovementioned study was a single-dose study, with no examination of how spermatogenesis recovers over time and after multiple treatment cycles. Thus, it is clinically important to evaluate spermatogenesis recovery following ifosfamide treatment in humans.

In our study, sperm parameters during cryopreservation were notably favorable compared to other reported diseases (Yumura et al., 2018). We also analyzed 12 patients who underwent semen analysis after treatment. In all these cases, ifosfamide was the key drug used, and spermatogenesis recovery was observed in 8/12 patients. However, among the four cases where sperm did not appear in the ejaculate, one patient passed away due to disease progression after a semen analysis 1 year post-cryopreservation, and another had no sperm in the semen analysis 1-year post-cryopreservation, subsequently being lost to follow-up. Hence, many patients who underwent semen analysis after chemotherapy experienced spermatogenesis recovery, with seven out of eight patients choosing to discard their cryopreserved sperm, except for one who passed away during the disease. Remarkably, spontaneous pregnancies and live births were achieved by two patients who had received 7 and 12 courses of regimens containing ifosfamide, respectively, suggesting that spermatogenesis may recover even with extended treatment durations. However, the patient cohort was too small to draw any conclusions, and there were cases of prolonged azoospermia (even following short treatment cycles) and poor prognosis requiring immediate multidisciplinary treatment. Consequently, there remains a great need for sperm cryopreservation for fertility. Use of doxorubicin-based chemotherapy regimens as alternatives to ifosfamide for malignant soft tissue tumors may induce testicular histopathological deformities, oligozoospermia, and abnormal sperm morphology. These drugs can affect the physiological role of Leydig and Sertoli cells, potentially causing chromosome abnormalities and DNA synthesis disruptions (Mohan et al., 2021b).

However, one patient who did not experience spermatogenesis recovery achieved pregnancy and live birth through ICSI using cryopreserved sperm during the first blastocyst transfer. This also underscores the critical importance of sperm cryopreservation for fertility preservation before chemotherapy. Given that many malignant BSTT patients are younger, clinicians should present the option of sperm cryopreservation for future family planning with thorough informed consent (Tozawa et al., 2022). The prognosis for malignant BSTT remains unfavorable (2), necessitating prompt treatment. Hence, the seamless preservation of fertility becomes imperative. To achieve this, orthopedic oncologists and reproductive health specialists should be aware of the need to devote sustainable resources to improve accessibility (Bedoschi and Navarro, 2022) by addressing factors like patient navigation, psychological supports, and administrative and financial supports. Establishment of regional networks may also help facilitate seamless collaboration between facilities (Furui et al., 2016).

This study presents several limitations. First, it was conducted at a single institution, and the sample size was relatively small. Malignant BSTT is a rare disease, and within this patient group, the subset of individuals considered for fertility preservation at a single institution was limited. Consequently, it is crucial to accumulate and analyze national data from multiple centers in Japan to enhance the robustness of the findings.

Second, the number of patients who requested semen testing was relatively low, which hindered a more precise assessment of spermatogenesis recovery after treatment. This could be attributed, in part, to the fact that many young patients may not opt for semen analysis because they do not currently intend to start a family. It would have been beneficial to incorporate semen analysis as part of routine follow-up outpatient care, ensuring regular checkups to provide a more comprehensive evaluation.

## 5 Conclusion

This study represents the first dedicated examination of spermatogenic function before and after chemotherapy for malignant BSTT. Despite the gonadotoxic properties of ifosfamide, it is noteworthy that spermatogenesis made a remarkable recovery in numerous cases. However, given prolonged azoospermia in specific instances, the importance of sperm cryopreservation before treatment cannot be overstated. Furthermore, it underscores the necessity for seamless collaboration between orthopedic oncologists and reproductive specialists to preserve fertility.

## Data Availability

The raw data supporting the conclusion of this article will be made available by the authors, without undue reservation.
